# Supplementary material for: PINK1 regulated mitophagy is evident in skeletal muscles
Source: Autophagy Rep. 2024 Mar 11;3(1):2326402. doi: 10.1080/27694127.2024.2326402 (PMC7616148; doi:10.1080/27694127.2024.2326402)
Supplement: Supplementaty files R1.docx [file KAUO_A_2326402_SM8920.docx]

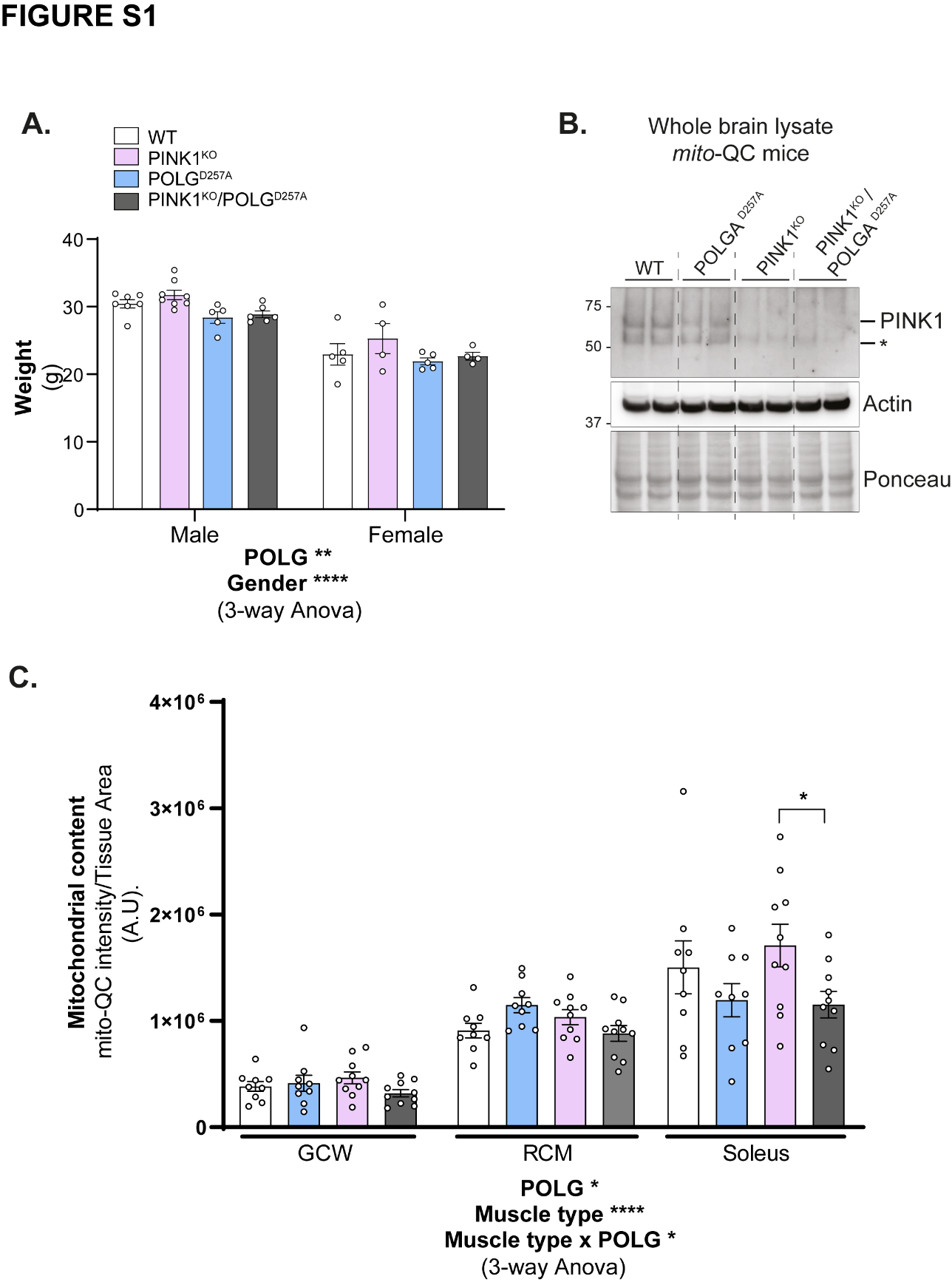


**Figure S1.** (A) Body weight of 180 days old mice of WT, PINK1 knock-out, mutator and double mutant (PINK1^KO^/POLG^D257A^) *mito*-QC mice (n=10-12 per group). Males and females were separated to better visualize the data. (B) Immunoblot of PINK1 immunoprecipitation from whole brain lysates of the same 4 groups (n=2 per group). Asterisk indicates an unspecific band. (C) Relative mitochondrial content in each muscle phenotype determined using the *mito*-QC GFP expression. (n=9-10). Overall data is represented as mean +/- SEM. Statistical significance of the main effects, and interaction effects of the 3-way ANOVAs are displayed below each graph, while results of the post-tests are displayed above the columns being compared. Statistical significance is displayed as *p<0.05, **p<0.01, and ****p<0.0001.

**
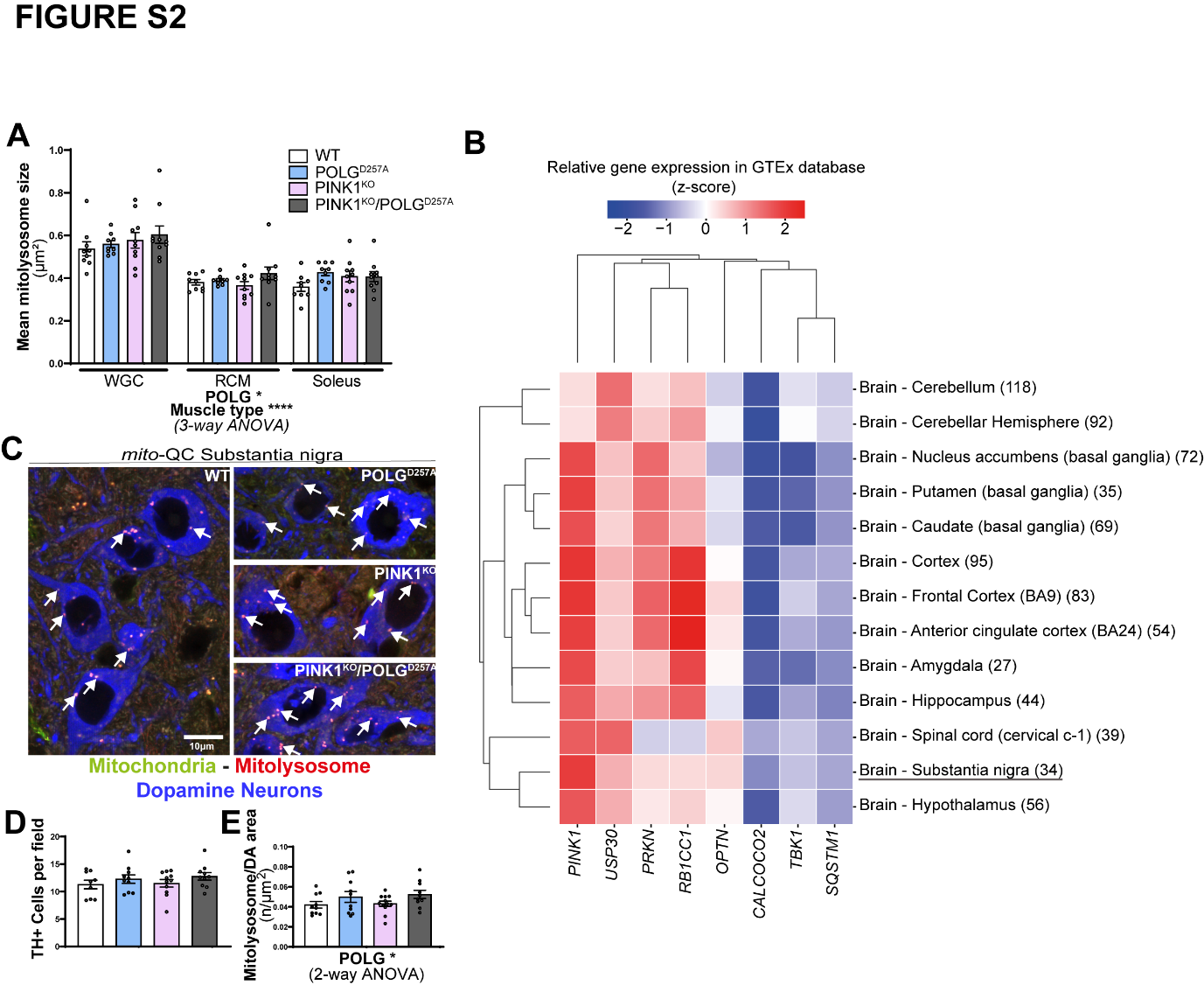
**

**Figure S2.** (A) Quantitation of basal mitophagy expressed by average size of a mitolysosome in the different areas of the skeletal muscles. (B) Gene-expression RNA-seq data analysis of the PINK1/Parkin pathway in different human brain regions, obtained from GTEx (Genotype-Tissue Expression). Numbers in brackets after each gene name indicate the number of independent samples. (C) Representative micrographs of tyrosine hydroxylase (TH) immunolabelled dopaminergic neurons of the substantia nigra pars compacta in WT, PINK1 knock-out, mutator and double mutant (PINK1^KO^/POLG^D257A^) *mito*-QC mice. Scale bar: 10 µm. White arrows indicate examples of mitolysosomes. (D) Quantitation of the number of TH positive cells per field of view using a 63x objective. (E) Quantitation of basal mitophagy in the DA neurons of the SNpc expressed as the number of mitolysosomes per TH positive area, (n=10-12). Overall data is represented as mean +/- SEM. Statistical significance of the main effects of the 2-way or 3-way ANOVAs are displayed below each graph, while results of the post-tests are displayed above the columns being compared. Statistical significance is displayed as *p<0.05, and ****p<0.0001.
